# Comparison of treatment outcome between glucocorticoids and non-steroidal anti-inflammatory drugs in subacute thyroiditis patients—a systematic review and meta-analysis

**DOI:** 10.3389/fendo.2024.1384365

**Published:** 2024-04-23

**Authors:** Anqi Yuan, Jialu Wu, Hui Huang

**Affiliations:** Department of Endocrinology and Metabolism, West China Hospital, Sichuan University, Chengdu, China

**Keywords:** subacute thyroiditis, treatment outcome, permanent hypothyroidism, recurrence rate, meta-analysis

## Abstract

**Importance:**

Subacute thyroiditis (SAT) is a self-limiting and inflammatory thyroid disease. Although SAT usually improves on its own within weeks, it needs treatment when patients have pain, fever, and symptoms of thyrotoxicosis. Therapeutic drugs mainly include non-steroidal anti-inflammatory drugs (NSAIDs) and glucocorticoids. Currently, there is no systematic review or meta-analysis of the comparison of outcomes between NSAIDs and glucocorticoids for the treatment of SAT.

**Objectives:**

To conduct a systematic review and meta-analysis on the outcomes in subacute thyroiditis patients treated with glucocorticoids or NSAIDs.

**Data sources:**

Using the four electronic databases, including PubMed, Embase, Cochrane Library, Wanfang database and Web of Science. All publications until 21 June 2023 were searched. The reference lists of all selected articles were independently screened to identify additional studies left out in the initial search.

**Study selection:**

The literature comparing outcomes between glucocorticoids and non-steroidal anti-inflammatory drugs for patients with subacute thyroiditis will be included.

**Data extraction and synthesis:**

Two independent investigators (Anqi Yuan and Jialu Wu) extracted the data following Preferred Reporting Items for Systematic Reviews and Meta-analyses guidelines (PRISMA) and then evaluated the quality of the eligible studies with the Newcastle-Ottawa Scale. Fixed-effects models for the meta-analyses were applied. Heterogeneity was assessed with the chi-squared (x²) test (Cochran’s Q) and inconsistency index (I²). The robustness of the results was tested with the sensitivity analyses. The bias of publication was assessed with the Harbord test.

**Main outcomes and measures:**

The incidence of permanent hypothyroidism in SAT patients treated with corticosteroids or NSAIDs.

**Results:**

Our study included a total of ten comparative cohort studies with 1337 participants. We found that the incidence of developing permanent hypothyroidism in the SAT patients who received glucocorticoids treatment was significantly lower than those who received NSAIDs treatment. (OR, 0.56; 95% CI, 0.36–0.88; P = 0.01). The risk of permanent hypothyroidism in patients who received prednisone at an average initial dose < 40 mg/d was significantly lower than that in patients who received NSAIDs (OR, 0.37; 95% CI, 0.14–0.94; P = 0.04). There was no significant difference in the occurrence of permanent hypothyroidism between SAT patients who received an average initial dose ≥ 40 mg/d of prednisone and those who received only NSAIDs (OR, 0.7; 95% CI, 0.14–3.53; P = 0.67). In addition, the recurrence rate was observably higher in those receiving glucocorticoids than in those receiving NSAIDs (OR, 1.98; 95% CI, 1.12–3.5; p = 0.02). The recurrence rate was significantly higher in patients with an average initial prednisone dose of < 40 mg/d than in the NSAIDs group. There was no significant difference in the recurrence rate between patients in the mean initial prednisone dose ≥ 40 mg/d group and those in the NSAIDs group.

**Conclusions and relevance:**

In this meta-analysis, we compared the treatment outcomes of SAT patients between glucocorticoids and NSAIDs. Our results indicated that glucocorticoid treatment was associated with a lower incidence of permanent hypothyroidism than NSAID treatment. Patients treated with NSAIDs might have a lower recurrence rate. This finding might help to understand the outcome of the disease when choosing different drugs and help physicians to make appropriate decisions.

**Systematic review registration:**

https://www.crd.york.ac.uk/prospero/, identifier CRD42023427332.

## Introduction

Subacute thyroiditis (SAT) is a self-limiting and inflammatory thyroid disease, accounting for approximately 5% of all thyroid diseases. SAT often occurs after an upper respiratory tract infection and is probably caused by a viral infection (1–4). The potentially causative viruses include Coxsackie virus, Echovirus, adenovirus, influenza virus, mumps and rubella virus, parvovirus B19, orthomyxovirus, HIV, Epstein-Barr virus, hepatitis E, measles virus, and the pandemic-spreading severe acute respiratory syndrome-coronavirus-2 (SARS-CoV-2) since the outbreak of novel coronavirus (COVID-19) in 2019 (5–7). Regarding pathogenesis, genetic susceptibility is related to SAT (7, 8), and previous studies have indicated that HLA-B* 35 is present in up to 70% of patients with SAT (9, 10). Besides, some drugs that affect the immune system may be associated with SAT, such as interferon for chronic hepatitis B or hepatitis C (11, 12). The high incidence of SAT is in 40–50– year– old women (3, 8), accounting for 75–80% of all SAT patients (7, 13). The typical clinical manifestations of SAT include fever, fatigue, thyroid pain, and symptoms of thyrotoxicosis. Blood tests show an elevated erythrocyte sedimentation rate (ESR), mild anemia, and increased white blood cell count (WBC). Thyroid ultrasound presents some typical signs like hypoechoic, ill-defined area with absent blood flow, and reduced uptake of radioactive iodine can also be observed by thyroid scintigraphy (14–17). The thyroid hormonal tests often show low thyroid stimulating hormone (TSH) and elevated free thyroxine (FT4) in the early stage of the disease. Sometimes, the diagnosis of subacute thyroiditis relies on pathological results of fine needle aspiration biopsy (FNAB) (7, 16, 18). Because of the nonspecific clinical symptoms in the early stage of SAT, it is easily misdiagnosed or delayed in diagnosis (19).

Non-steroidal anti-inflammatory drugs (NSAIDs) and glucocorticoids are always recommended for the treatment of SAT to relieve symptoms and mitigate inflammation (20). The clinical guidelines of the American Thyroid Association (ATA) suggest that patients with mild symptomatic SAT should initially be treated with β-adrenergic-blocking drugs and NSAIDs to control thyrotoxic symptoms or relieve pain. For patients with moderate to severe pain or thyrotoxic symptoms, corticosteroids are administered with the standard recommended dosage of prednisone 40 mg daily for a duration of 1 to 2 weeks, then the dosage is gradually decreased according to the clinical response until withdrawal (16). Up to now, a certain number of studies have explored the different therapeutic effects of different drugs or various dosages of the same drugs in patients with SAT, which include duration of symptom relief time, duration of thyroid function recovery, recurrence rate, and adverse reactions. Some studies have indicated that glucocorticoids were quicker than NSAIDs in relieving symptoms and restoring normal thyroid function in SAT patients (21). However, there are no related clinical guidelines on how to choose NSAIDs or glucocorticoids when treating SAT. SAT is a self-limiting disease characterized by three phases: the initial thyrotoxic phase, the transient hypothyroidism phase, and the recovery stage in which thyroid function returns to normal (16, 22, 23). However, the occurrence of permanent hypothyroidism has been documented in several studies, with rates ranging from 5% to 26% (18), in which some studies have indicated a higher incidence of permanent hypothyroidism in cohorts treated with glucocorticoids (15). In contrast, other studies’ findings suggested that the incidence of this condition did not differ between groups receiving NSAIDs and groups receiving glucocorticoids (24). Furthermore, the recurrence has been reported during follow-up in the last 20 years, with approximately 6.9% to 22.2% (25, 26). Whether the development of permanent hypothyroidism and recurrence is associated with the different drug treatments and doses of SAT is still ambiguous.

Therefore, we conducted the first systematic review and meta-analysis to compare treatment outcomes between glucocorticoids and NSAIDs in SAT patients, in which we placed great emphasis on the incidence of permanent hypothyroidism after the two different treatments. It aims to assist clinicians in guiding clinical decisions.

## Methods

This systematic review and meta-analysis followed the Preferred Reporting Items for Systematic Reviews and Meta-analyses (PRISMA) statement 21 and was registered in the PROSPERO database (identifier: CRD42023427332).

### Literature search

We performed a comprehensive literature search of PubMed, Embase, Cochrane Library, Chinese Wanfang database, and Web of Science from the inception of the databases to June 21, 2023, to identify studies comparing treatment outcomes between glucocorticoids and NSAIDs in SAT patients, without any language restrictions. We also hand-screened the reference lists of primary cited articles to ensure access to articles that were not captured by electronic retrieval. We searched the databases based on the following MeSH terms: “Glucocorticoid”, “Anti-Inflammatory Agents, Non-Steroidal”, and “Thyroiditis, Subacute”. The detailed search strategy is described in **Supplementary S1**. Two investigators (Anqi Yuan and Jialu Wu) independently screened titles and abstracts based on selection criteria and reviewed the full-text articles obtained from the initial retrieval.

### Selection criteria

We included cohort studies that met the following criteria for this meta-analysis: (i) human-based observational studies (cohort studies and case-control studies) or randomized controlled trials; (ii) the subjects were adults who were definitely diagnosed with SAT by typical clinical symptoms, laboratory data, radioactive iodine uptake (RAIU) test/thyroid scintigram and/or thyroid ultrasound; (iii) the treatment outcomes of steroids and NSAIDs in SAT patients were compared, and the outcome measures included permanent hypothyroidism or recurrence rate. Any study that met one of the following criteria was excluded by us: (i) Studies without a comparison of glucocorticoids and non-steroidal anti-inflammatory drugs treatment; (ii) No outcome measure for permanent hypothyroidism and recurrence rate was presented; (iii) Letters, editorial comments, case reports, conference abstract, meta-analyses, review, and patents. We defined permanent hypothyroidism as the presence of hypothyroidism (including overt hypothyroidism and subclinical hypothyroidism) requiring levothyroxine replacement at six months after SAT. Recurrence is defined as the reappearance of clinical symptoms during drug dose reduction or after discontinuation, with elevated ESR/CRP.

### Data extraction

Data extraction was carried out independently by two investigators (Anqi Yuan and Jialu Wu), and disagreements were resolved by discussion with a third investigator. We extracted the following information by reviewing the full texts of the included studies: the basic information (first author, year of publication, country of study, study design, duration of the study), information on the study cohorts (sample size, age, type of treatment, starting drug dose, treatment duration, follow-up duration), therapeutic effects of two drugs (NSAIDs and glucocorticoids), and outcome indicators (number of permanent hypothyroidism and recurrence).

### Quality assessment

Two investigators separately assessed the quality of the included studies via the Newcastle -Ottawa Scale (NOS) (27) for cohort studies, and the discrepancies were resolved through discussion. The NOS primarily consists of three dimensions, including selection of study groups, comparability of groups, and ascertainment of outcomes (cohort studies) or exposure (case-control studies), each with a total score of 4, 2, and 3 points. The NOS scores range from 0 to 9, with higher scores indicating less risk of bias. The scores of the three domains were summed, and studies with a total score ≥ 7 were considered to have a low risk of bias (28).

### Statistical analysis

The odds ratio (OR) was applied for the comparison of dichotomous variables. In literature data collection, continuous variables conforming to a normal distribution were expressed as mean ± standard, and non-normally distributed variables were expressed as median (range) values. In the meta-analysis, we pooled OR and 95% confidence intervals (CIs) of individual studies. Heterogeneity was assessed with the chi-squared (x²) test (Cochran’s Q) and inconsistency index (I²). In the heterogeneity test, when the p-value > 0.05 and the clinical and methodological heterogeneity among the included studies were small, the fixed effect model was adopted; otherwise, the random effect model was employed. Since our study was reviewed and screened in strict accordance with the inclusion and exclusion criteria, the subjects were all adult patients with clinically confirmed SAT without significant differences in age, intervention measures, and outcome indicators. Combined with the p-value of the heterogeneity test, we chose the fixed effect model with the statistical method of Mantel-Hasenzel. In addition, we used the Galbraith plot to visually assess heterogeneity caused by individual studies. The results of the meta-analysis are presented as forest plots. To evaluate the publication bias of the included articles, we used the Harbord test. A sensitivity analysis using the leave-one-out method was performed to assess the robustness of the results. All analyses were performed with STATA version 17 and Review Manager version 5.4.

## Results

### Literature search and study characteristics

**Figure 1:**
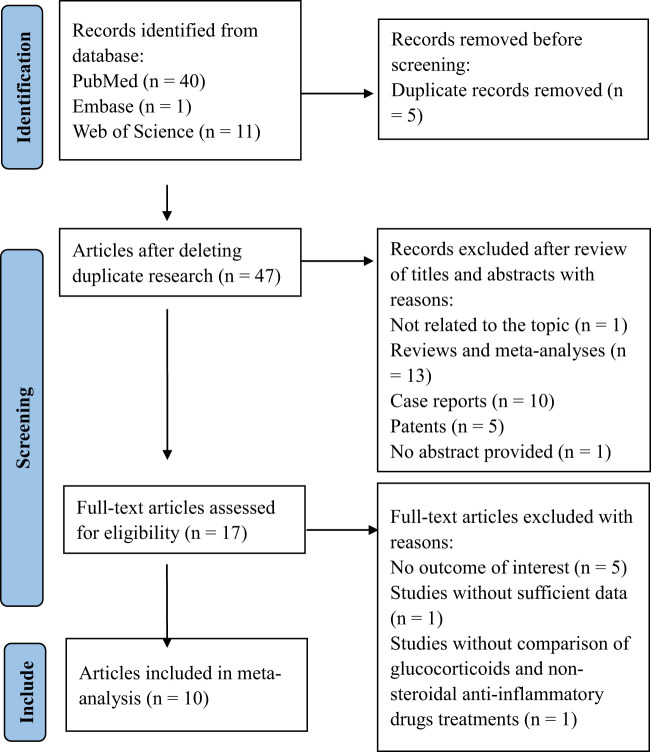
PRISMA flow diagram of the systemic review procedure,.

The flow diagram of the literature search and selection process is shown in **Figure 1**. According to the search strategy, a total of 48 related records were retrieved from PubMed (n = 40), Embase (n = 1), and Web of Science (n = 11) databases. After deleting duplicate articles, forty-seven articles were reviewed for titles and abstracts. Thirty articles were excluded due to their inappropriate article types. Then, seventeen publications were screened for full text in detail, and seven publications were excluded. Among the excluded articles, five were eliminated due to the absence of outcome measures for permanent hypothyroidism or recurrence, one was excluded for insufficient data, and another was excluded because it failed to compare the treatment effectiveness of glucocorticoids and NSAIDs. Eventually, ten studies involving 1337 patients were eligible and included in the meta-analysis (13, 15, 21, 24, 29–34). All ten included studies were cohort studies (nine were retrospective, and one was prospective). The main features of the included studies are presented in **Table 1**.

**Table 1:** Characteristics of studies included in the meta-analysis.

						glucocorticoids				NSAIDs			
Authors	Country	Study period	Mean age, y	Study design	Mean follow-up, mo	Total, n	Initial therapeutic type and average daily dose	Permanent hypothyroidism, n	Duration of therapy, d	Total, n	Initial therapeutic type and average daily dose	Permanent hypothyroidism, n	Duration of treatment, d
Sencar et al., 2019 (29)	Turkey	2014-2018	43 ± 9	Retrospective study	27 (6.2–64)	159	48 mg methylprednisolone	14	42(14–70)	57	1800mg ibuprofen	13	14 (2-42)
Saydam et al., 2022 (30)	Turkey	2017-2019	45.2 ± 11.1	Retrospective study	36	37	18 were initiated 33.92 (± 7.18) mg prednisolone; 19 were initiated 15 mg.	3(from 15 mg/d)	0.5 mg/kg: 48 (42-126); 15mg: 42 (42-59)	26	1000 mg Naproxen sodium	8	–
Saklamaz, 2017 (31)	Turkey	2010-2015	–	Retrospective study	12	48	–	10	–	33	–	5	–
Nishihara et al., 2009 (32)	Japan	1996-2004	48.6 ± 9.5	Retrospective study	21.1 ± 17.3	158	17.5 mg prednisone	7	42	48	–	3	–
Fatourechi et al., 2003 (15)	Minnesota	1960-1997	45 (14-87)	Retrospective study	–	34	40 mg prednisone	8	34	39	–	6	–
Benbassat et al., 2007 (24)	Israel	1999-2005	48.6 ± 12	Retrospective study	12	18	–	2	–	25	–	3	–
Zhao et al., 2020 (33)	China	2015-2017	44.07 ± 9.20	Prospective study	24	13	prednisolone	1	–	46	–	9	–
Erdem et al., 2007 (13)	Turkey	1987-2001	34.0 ± 17.8	Retrospective study	60	50	35-60 mg prednisolone	–	–	119	–	–	–
Sato et al., 2014 (21)	Japan	2008-2014	48.8 ± 12.8	Retrospective study	–	25	15 mg prednisolone	–	–	17	180 mg loxoprofen	–	–
Bahadir et al., 2022 (34)	Turkey	2008-2022		Retrospective study	12	72	32 mg methylprednisolone	–	at least 56 days	65	800-1200 mg ibuprofen	–	at least 56 days

The quality assessment of the included studies with the NOS score is described in **Table 2**. The average NOS score was 8.4, and nine studies were considered high-quality with a low risk of bias. None of the ten studies received 2 scores in the “comparability” dimension of the NOS. Two studies did not get full marks in the “outcome” section because of a higher proportion of lost follow-up.

**Table 2:** Quality evaluation of the eligible studies with the Newcastle-Ottawa Scale (range 0-9).

Study	Representativeness of the exposed cohort	Selection of the nonexposed cohort	Ascertainment of exposure	Outcome not present at the start	Comparability on the most important factor	Assessment of outcome	Long enough follow-up(≥1 year)	Adequacy of follow-up of cohorts	Quality score
Sencar	*	*	*	*	*	*	*	–	7
Saydam	*	*	*	*	*	*	*	*	8
Saklamaz	*	*	*	*	*	*	*	*	8
Nishihara	*	*	*	*	*	*	*	*	8
Fatourechi	*	*	*	*	–	*	*	–	6
Benbassat	*	*	*	*	*	*	*	*	8
Zhao	*	*	*	*	*	*	*	*	8
Erdem	*	*	*	*	*	*	*	*	8
Sato	*	*	*	*	*	*	*	–	7
Bahadir	*	*	*	*	*	*	*	*	8

Each asterisk (*) represents 1 score.

### Comparing the incidence of permanent hypothyroidism of treatment with glucocorticoids versus NSAIDs

**Figure 2:**
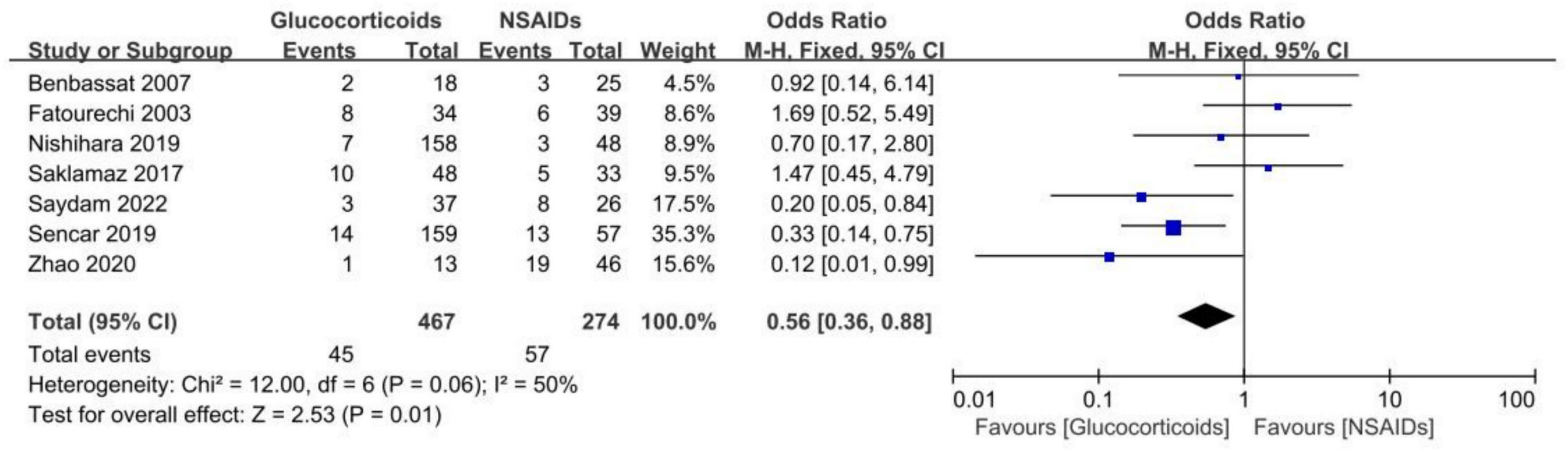
Forest plot showing the meta-analysis of permanent hypothyroidism risk in glucocorticoids versus NSAIDs.

**Figure 3:**
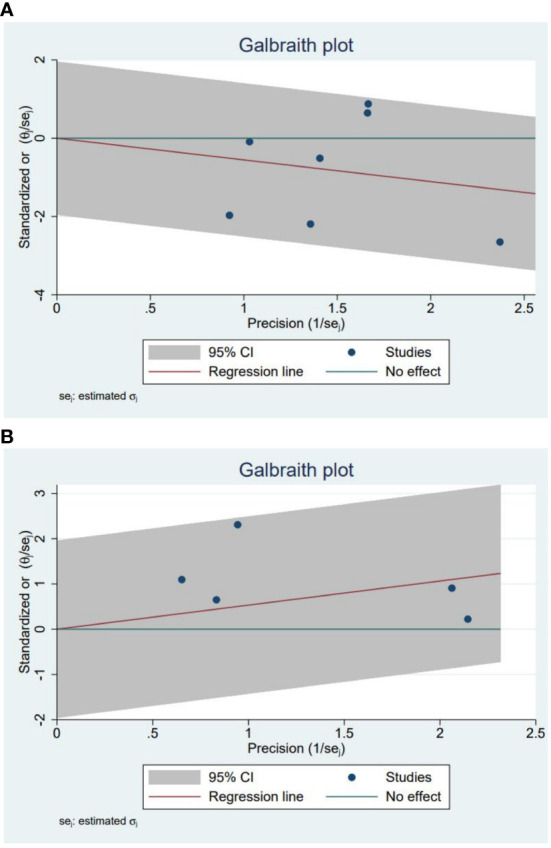
**(A)** The Galbraith plot for the incidence of permanent hypothyroidism in glucocorticoids versus NSAIDs. **(B)** The Galbraith plot for the recurrence in glucocorticoids versus NSAIDs.

The purpose of our study was to compare treatment outcomes between glucocorticoids and NSAIDs in SAT patients with the primary outcome indicator of permanent hypothyroidism. A total of seven studies involving 741 cases reported SAT treatment outcomes of permanent hypothyroidism (15, 24, 29–33). Based on the seven studies, our study revealed that the odds ratio of developing permanent hypothyroidism was significantly lower with glucocorticoids treatment compared to NSAIDs treatment in SAT patients (OR, 0.56; 95% CI, 0.36–0.88; P = 0.01), as presented in **Figure 2**. Patients treated with NSAIDs may have more than 40% higher risk of developing permanent hypothyroidism. There was moderate heterogeneity among the studies (I² = 50%, p = 0.06). Meanwhile, the Galbraith plot was also employed to explore the heterogeneity of the seven studies, and the results displayed all studies were within two standard deviations from the regression line, indicating no significant heterogeneity (**Figure 3A**).

**Figure 4:**
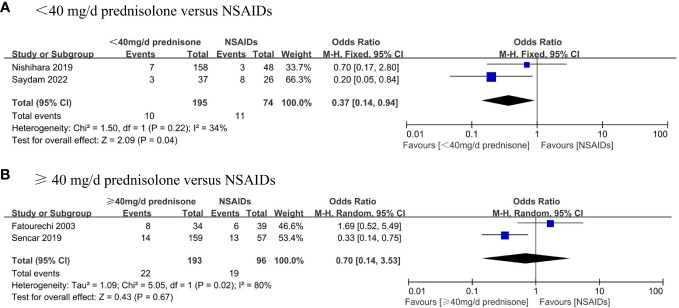
Forest plot showing the meta-analysis of permanent hypothyroidism risk in different dosage glucocorticoids versus NSAIDs. **(A)**, < 40 mg/d prednisone versus NSAIDs. **(B)**, ≥ 40 mg/d prednisone versus NSAIDs.

Our search also performed subgroup analyses that were pre-specified and aimed to assess the incidence of permanent hypothyroidism in different dosage glucocorticoid treatments and NSAID treatments. Four studies described the exact dosage of prednisone used in detail. We divided the SAT patients were divided into two groups based on whether the mean initial dose of prednisone exceeded 40 mg daily. We compared the outcome with that using NSAIDs respectively. We found that the incidence of permanent hypothyroidism in the group of prednisone dose < 40 mg/d was significantly lower than in the patients taking NSAIDs (OR, 0.37; 95% CI, 0.14–0.94; P = 0.04) with mild heterogeneity (I² = 34%, p = 0.22), as shown in the **Figure 4A**. Besides, there was no significant difference in the occurrence of permanent hypothyroidism between the group of prednisone dose ≥ 40 mg/d and that using NSAIDs (OR, 0.7; 95% CI, 0.14–3.53; P = 0.67) (**Figure 4B**).

### Comparing the recurrence rate of SAT patients treated with glucocorticoids versus NSAIDs

**Figure 5:**
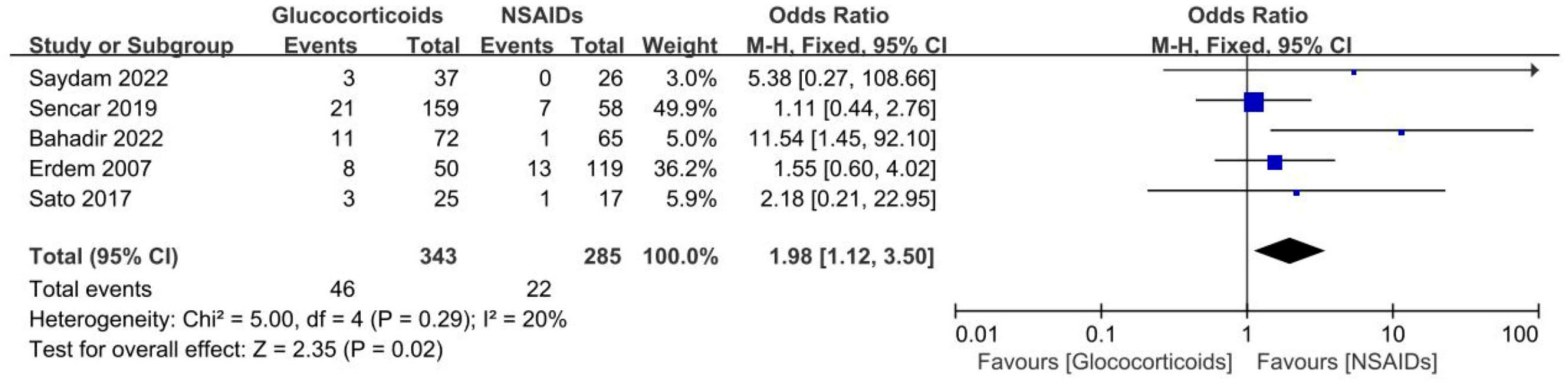
Forest plot showing the meta-analysis of recurrence in glucocorticoids versus NSAIDs.

Five studies involving 628 SAT patients treated with glucocorticoids or NSAIDs evaluated the recurrence rates of SAT (13, 21, 29, 30, 34). The analysis demonstrated that the recurrence rate was observably higher in the patients using glucocorticoids than in those using NSAIDs (OR, 1.98; 95% CI, 1.12–3.5; p = 0.02) without significant heterogeneity (I² = 20%, p = 0.29), as summarized in **Figure 5**. For patients with SAT, the risk of recurrence with glucocorticoids was approximately twice as high as with NSAIDs. Furthermore, the Galbraith plot confirmed the absence of significant heterogeneity among the five studies included (**Figure 3B**).

**Figure 6:**
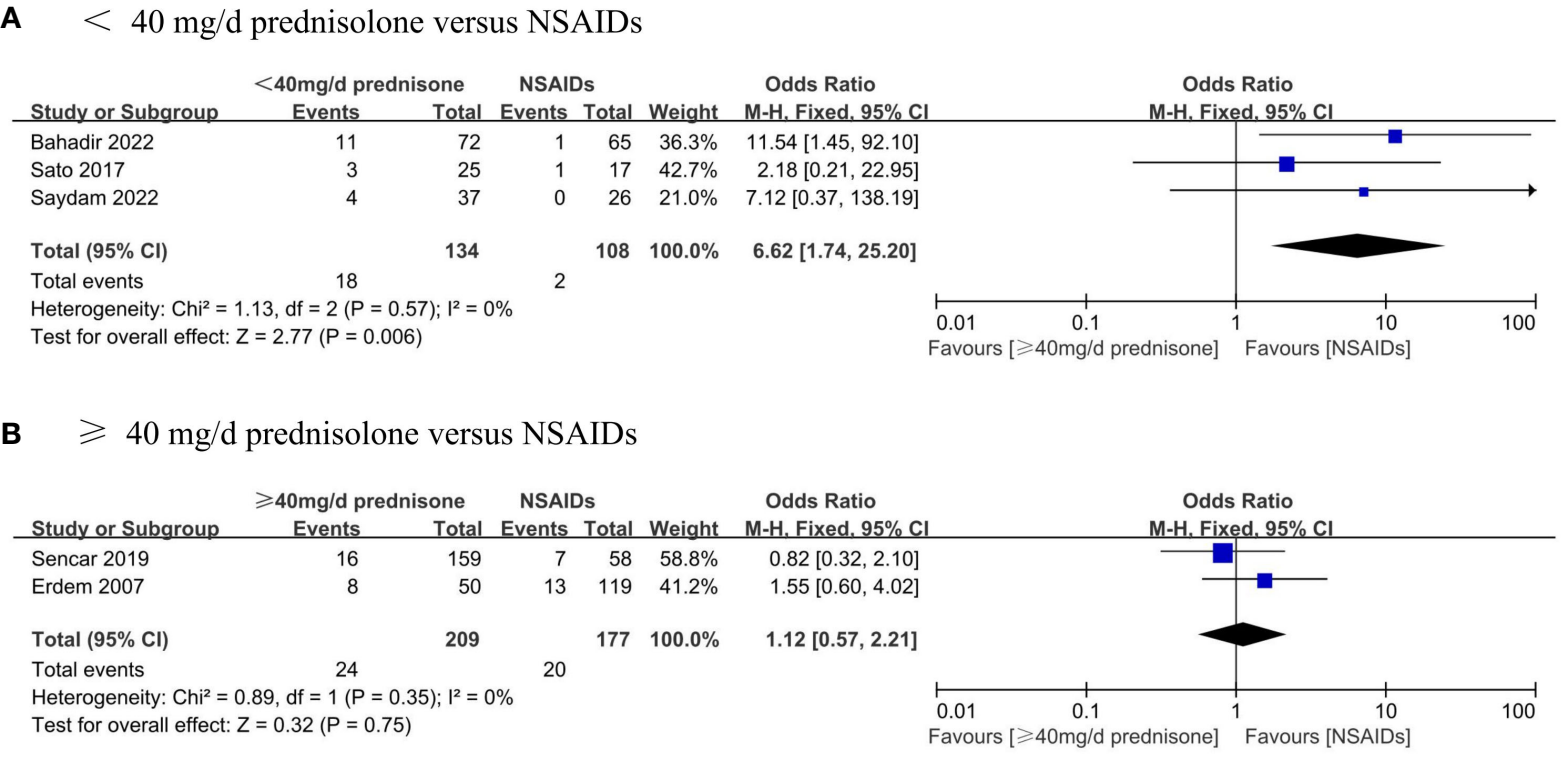
Forest plot showing the meta-analysis of recurrence rate in different dosage glucocorticoids versus NSAIDs. **(A)**, < 40 mg/d prednisone versus NSAIDs. **(B)**, ≥ 40 mg/d prednisone versus NSAIDs.

Furthermore, subgroup analyses were performed to assess the recurrence rate between different doses of glucocorticoids and NSAIDs. Patients were divided into two groups based on whether they received an average initial dose of prednisone exceeding 40 mg/d. The results displayed that the recurrence rate of patients in the prednisone < 40 mg/d group was significantly higher than that in the NSAIDs group (OR, 6.62; 95% CI,1.74–25.2; P = 0.006) without significant heterogeneity (I² = 0%, p = 0.57) (**Figure 6A**). There was no significant difference in the recurrence rate between patients in the ≥40 mg/d prednisone group and those in the NSAIDs group (OR, 1.12; 95% CI, 0.57–2.21; P = 0.75) (**Figure 6B**).

### Publication bias and sensitivity analysis

We conducted the Harbord test to explore the publication bias of included studies. Concerning the incidence of permanent hypothyroidism, the results of the Harbord test indicated no significant publication bias (P = 0.54). The Harbord test for recurrence rate also revealed no significant publication bias (P = 0.21). The leave-one-out sensitivity analysis was conducted to determine the stability of the results. In the meta-analysis comparing the incidence of permanent hypothyroidism between patients treated with glucocorticoids and NSAIDs, the removal of three high-weight studies separately did not significantly alter the heterogeneity, but it did change the combined effect size. The lowest summary odds ratio was 0.45 (leaving out the study by Fatourechi), while the highest was 0.69 (leaving out the study by Sencar), indicating a lack of stability in the results and need to be interpreted with caution. Regarding the recurrence rates between the groups, the omittance of only the study by Bahadir resulted in a significant change in the combined effect size, showing mild instability of the results.

## Discussion

Glucocorticoids and NSAIDs are considered the common drugs in the treatment of SAT to anti-inflammatory and relieve symptoms. The clinical guideline of the American Thyroid Association (ATA) recommends NSAIDs preferentially for patients with mild degree and glucocorticoids preferentially for patients with moderate to severe degrees (16). Yet, so far, there is no clear and complete definition of mild, moderate, and severe SAT patients. In recent years, the number of SAT patients has been increasing, and the importance of its standardized treatment cannot be ignored. We performed the first meta-analysis of the treatment of subacute thyroiditis to review the treatment outcomes between the patients with glucocorticoids and other patients with NSAIDs. Our results demonstrated that the risk of permanent hypothyroidism was higher with NSAIDs treatment than with glucocorticoids treatment, and the recurrence rate is higher in patients treated with glucocorticoids compared to treated NSAIDs. Furthermore, our subgroup analysis found that the incidence of permanent hypothyroidism in the patients accepted < 40 mg/d prednisone was lower than those accepted NSAIDs.

At present, the etiology and pathogenesis of subacute thyroiditis are not completely clear. The published literature generally deems that its pathogenic mechanism includes genetic susceptibility related to human leukocyte antigen, predisposing factors related to viral infection, and immune disorders (7, 34). The virus infection may lead to immune disorders, including excessive immune response and immune deficiency, as well as direct damage to thyroid cells (35). Besides, during the COVID-19 pandemic, the SARS-CoV-2 coronavirus can trigger an overactive immune response and an increase in interleukin-6 levels, potentially leading to thyroiditis. Immune disorders play an important role in the occurrence of SAT. Glucocorticoids have the pharmacological effects of anti-inflammatory, antivirus, and immunosuppression. Prednisone, as a medium-acting preparation of glucocorticoids, is commonly applied in treating subacute thyroiditis. NSAIDs have anti-inflammatory, antipyretic, and analgesic pharmacological effects, but their anti-inflammatory effects are not as good as prednisone (21).

Our study found the incidence of permanent hypothyroidism was significantly higher in patients treated with NSAIDs than in those treated with glucocorticoids. The mechanisms for the development of permanent hypothyroidism of SAT have not been fully elucidated. Gzariu et al. pointed out that marked fibrosis in thyroid glands after the course of SAT might be one of the mechanisms (36). A retrospective study involving 95 Chinese patients with SAT found that patients with higher levels of C-reactive protein (CRP) and lower levels of thyroid-stimulating hormone (TSH) were more prone to developing hypothyroidism. Therefore, thyroid function should be closely monitored, especially in patients with CRP > 97.80 mg/L and TSH < 0.10 mIU/L (37). The higher CRP levels may indicate more severe inflammation that can damage the thyroid gland and result in hypothyroidism (37). On the contrary, Omori et al. found no significant association between CRP and permanent hypothyroidism (38). Simultaneously, they suggested that the hypoechoic area in the entire thyroid gland represented the extent of thyroid cell destruction associated with active inflammation but was not related to the development of permanent hypothyroidism (38). The study by Zhao et al. revealed that a higher early maximum TSH value within three months after SAT onset may be a risk factor for the incidence of hypothyroidism two years later (33). Sencar et al. reported that ibuprofen treatment and anti-thyroid peroxidase antibody (TPOAb) positivity were identified as risk factors for permanent hypothyroidism, aligning with expectations. The initial levels of TSH, ESR, and CRP were not detected as risk factors for permanent hypothyroidism (29). Görges et al. performed a retrospective study involving 127 SAT patients who received either NSAIDs or glucocorticoids. They reported that higher cumulative glucocorticoid doses were associated with a greater prevalence of hypothyroidism. Patients who received a total dose of more than 4 g of prednisolone were more likely to develop hypothyroidism later than those who received a lower cumulative dose of prednisolone. It is likely that patients who received a higher cumulative dose of prednisolone had a more severe course of SAT (18). The opinion was consistent with the study conducted by Fatourechi et al. (15). Nonetheless, the study by Benbassat showed that there was no difference in the incidence of permanent hypothyroidism between the group treated with NSAIDs and the group treated with glucocorticoids, although the glucocorticoids group had more severe clinical features (24). The variation in findings of these studies may be due to differences in the number of participants enrolled or geographic differences. Unfortunately, the study did not address the initial assignment of patients with permanent hypothyroidism (18). Zhao et al. noted that the thyroid gland volume of SAT patients with hypothyroidism was smaller than that of healthy controls (33). Schenke et al. indicated that SAT patients with permanent hypothyroidism had a smaller thyroid volume compared to SAT patients without hypothyroidism (17). A smaller residual thyroid volume could be one of the factors contributing to permanent hypothyroidism. A reduction in thyroid volume to less than 6 ml in women and less than 8 ml in men is considered evidence of permanent hypothyroidism (18).

Recurrence is also one of the notable long-term outcomes for patients with SAT. Our study manifested that the recurrence rate was significantly higher in patients who received glucocorticoids than in those who received NSAIDs. The result was in accordance with the study by Zhang et al. (39). The underlying cause of recurrence may be that glucocorticoids are used to treat severe patients or patients who do not respond to NSAIDs. These patients are more likely to experience SAT relapses. An alternative explanation is that glucocorticoids may have suppressed the inflammatory response without affecting the disease process and rebounded after the discontinuation of glucocorticoids (34). Moreover, one study reviewed potential predictors for the recurrence of SAT, including the *HLA* haplotype, the sialic acid level, the TSH level at the termination of treatment, further extension of the hypoechoic area, and an increase in thyroid volume. Their study also indicated that age and sex are not statistically significantly associated with SAT recurrence (39). Hepsen et al. found that a lower TSH level at the end of the treatment was a predictor of recurrence (40). Stasiak et al. revealed that the determining factor for SAT recurrence was the coexistence of HLA-B*18:01 and HLA-B*35. Moreover, they found that the non-recurrence group had lower TSH, higher FT4, and free triiodothyronine (FT3) levels than the recurrence group. Elevated TPOAb concentration at the first SAT episode was identified as a protective factor (41). The extent of thyroid tissue damage appears to play a vital role in the risk of recurrence. Similarly, Sencar et al. reported that the levels of FT4 were significantly higher in the non-recurrence group (29). Soltani et al. conducted a meta-analysis to identify the lowest effective initial dose of prednisolone for the treatment of SAT. They concluded that there was a significant correlation between the initial mean dose of prednisolone (PSL) and the recurrence rate. The recurrence rate was lower in the group with an initial average dose of prednisolone ≤ 20 mg/d than in the group with an initial average dose of prednisolone > 20 mg/d (42). Nevertheless, Arao et al. showed that the recurrence rate was not correlated with the initial dose of PSL. The number of days required to taper the PSL dose to 5 mg/d was significantly shorter in the non-relapse group compared to the relapse group (43). Additionally, it has been suggested that the harmful effects of steroids on viral replication and clearance could be one of the reasons for recurrence in SAT patients (29).

Our subgroup analysis exhibited that the incidence of permanent hypothyroidism in patients who accepted the average initial dose < 40 mg/d of prednisone was significantly lower than in patients who accepted NSAIDs, and there was no significant difference in the occurrence of permanent hypothyroidism between patients who received the average initial dose ≥ 40 mg/d of prednisone and those who received NSAIDs. In the subgroup analysis of recurrence rate, we found that the recurrence rate was significantly higher in patients with an average initial prednisone dose of < 40 mg/d than in the NSAIDs group. There was no significant difference in the recurrence rate between patients in the group of mean initial prednisone dose ≥ 40 mg/d and those in the NSAIDs group. However, the number of studies included in the subgroup analysis was small, and the heterogeneity among some studies was significant. Therefore, further studies are needed to determine the relationship between different doses of glucocorticoids and permanent hypothyroidism. In Turkey, a retrospective cohort study with up to 1 year of follow-up was performed. The study divided SAT patients into two groups: the 16 mg methylprednisolone (MPS) group and the 48 mg MPS group. The results indicated that the 48 mg MPS group had a higher SAT recurrence rate than the 16 mg MPS group. There was no significant difference in the rate of permanent hypothyroidism between the two groups (40). Koirala et al. recommend that 20 mg of prednisolone daily tapered over four weeks is appropriate (44). Our research indicates that glucocorticoid therapy appears to be a protective factor against the development of permanent hypothyroidism compared to NSAIDs. However, it is well known that long-term and high-dose usage of glucocorticoids may lead to some side effects, such as drug-induced Cushing’s syndrome, gastrointestinal symptoms, hypertension, diabetes, and osteoporosis. Thus, the efficacy and appropriateness of an initial low dose of glucocorticoids, but a slow tapering of the dose is necessary, which may prevent recurrence. At the same time, it is essential for us to closely monitor the adverse reactions caused by the use of glucocorticoids and achieve individualized treatment.

### Strengths and limitations

Our meta-analysis was the first to research the differences in treatment outcomes between SAT patients treated with glucocorticoids and those treated with NSAIDs. We conducted a comprehensive literature search, and the included studies were all cohort studies with long follow-ups and no severe heterogeneity among them. The topic of our study is clinically relevant and provides evidence of treatment outcomes of SAT for physicians and patients in order to make better clinical decisions. However, several limitations of our study cannot be ignored. First, most of the included studies in the analysis were retrospective, without randomized controlled trials and appropriate control of confounding factors, and the previous clinical data may have a certain degree of omission and uncertainty. In addition, sensitivity analyses were performed and showed a lack of stability, so the results of this combined analysis should be interpreted with caution. Finally, some of the included studies did not address the specific dosage and duration of medication. Thus, the outcomes with different drug doses could not be further examined. More large-scale, double-blind, prospective, multicenter randomized controlled studies with long-term follow-up are needed for a more robust and precise conclusion.

## Conclusion

Our meta-analysis demonstrated that subacute thyroiditis patients treated with NSAIDs may have a greater risk of permanent hypothyroidism compared to those treated with glucocorticoids. The recurrence rate of SAT might be higher in patients who require treatment with glucocorticoids. The potential predictors for the recurrence of SAT may be multifaceted. Due to the instability and bias of existing study results, further research is needed to guide physicians in making appropriate treatment plans for subacute thyroiditis.

## Data availability statement

The original contributions presented in the study are included in the article/**Supplementary Material**. Further inquiries can be directed to the corresponding author.

## Author contributions

AY: Data curation, Investigation, Writing – original draft. JW: Data curation, Investigation, Writing – review & editing. HH: Methodology, Supervision, Validation, Writing – review & editing.
