# Supplementary material for: Comparison of treatment outcome between glucocorticoids and non-steroidal anti-inflammatory drugs in subacute thyroiditis patients—a systematic review and meta-analysis
Source: Front Endocrinol (Lausanne). 2024 Apr 23;15:1384365. doi: 10.3389/fendo.2024.1384365 (PMC11075098; doi:10.3389/fendo.2024.1384365)
Supplement: Supplementary file 1 [file Table_1.docx]

**search strategy**

("thyroiditis, subacute"[MeSH Terms] OR ("thyroiditis, subacute"[MeSH Terms] OR ("thyroiditis"[All Fields] AND "subacute"[All Fields]) OR "subacute thyroiditis"[All Fields] OR ("thyroiditis"[All Fields] AND "subacute"[All Fields]) OR "thyroiditis subacute"[All Fields] OR ("thyroiditis, subacute"[MeSH Terms] OR ("thyroiditis"[All Fields] AND "subacute"[All Fields]) OR "subacute thyroiditis"[All Fields] OR ("subacute"[All Fields] AND "thyroiditides"[All Fields]) OR "subacute thyroiditides"[All Fields]) OR ("thyroiditis, subacute"[MeSH Terms] OR ("thyroiditis"[All Fields] AND "subacute"[All Fields]) OR "subacute thyroiditis"[All Fields] OR ("subacute"[All Fields] AND "thyroiditis"[All Fields])) OR ("thyroiditis, subacute"[MeSH Terms] OR ("thyroiditis"[All Fields] AND "subacute"[All Fields]) OR "subacute thyroiditis"[All Fields] OR ("thyroiditides"[All Fields] AND "subacute"[All Fields])) OR ("thyroiditis, subacute"[MeSH Terms] OR ("thyroiditis"[All Fields] AND "subacute"[All Fields]) OR "subacute thyroiditis"[All Fields] OR ("subacute"[All Fields] AND "painful"[All Fields] AND "thyroiditis"[All Fields]) OR "subacute painful thyroiditis"[All Fields]) OR ("thyroiditis, subacute"[MeSH Terms] OR ("thyroiditis"[All Fields] AND "subacute"[All Fields]) OR "subacute thyroiditis"[All Fields] OR ("painful"[All Fields] AND "thyroiditides"[All Fields] AND "subacute"[All Fields])) OR ("thyroiditis, subacute"[MeSH Terms] OR ("thyroiditis"[All Fields] AND "subacute"[All Fields]) OR "subacute thyroiditis"[All Fields] OR ("painful"[All Fields] AND "thyroiditis"[All Fields] AND "subacute"[All Fields]) OR "painful thyroiditis subacute"[All Fields]) OR ("thyroiditis, subacute"[MeSH Terms] OR ("thyroiditis"[All Fields] AND "subacute"[All Fields]) OR "subacute thyroiditis"[All Fields] OR ("subacute"[All Fields] AND "painful"[All Fields] AND "thyroiditides"[All Fields])) OR ("thyroiditis, subacute"[MeSH Terms] OR ("thyroiditis"[All Fields] AND "subacute"[All Fields]) OR "subacute thyroiditis"[All Fields] OR ("thyroiditides"[All Fields] AND "subacute"[All Fields] AND "painful"[All Fields])) OR ("thyroiditis, subacute"[MeSH Terms] OR ("thyroiditis"[All Fields] AND "subacute"[All Fields]) OR "subacute thyroiditis"[All Fields] OR ("thyroiditis"[All Fields] AND "subacute"[All Fields] AND "painful"[All Fields])) OR ("thyroiditis, subacute"[MeSH Terms] OR ("thyroiditis"[All Fields] AND "subacute"[All Fields]) OR "subacute thyroiditis"[All Fields] OR ("granulomatous"[All Fields] AND "thyroiditis"[All Fields]) OR "granulomatous thyroiditis"[All Fields]) OR ("thyroiditis, subacute"[MeSH Terms] OR ("thyroiditis"[All Fields] AND "subacute"[All Fields]) OR "subacute thyroiditis"[All Fields] OR ("granulomatous"[All Fields] AND "thyroiditides"[All Fields])) OR ("thyroiditis, subacute"[MeSH Terms] OR ("thyroiditis"[All Fields] AND "subacute"[All Fields]) OR "subacute thyroiditis"[All Fields] OR ("thyroiditides"[All Fields] AND "granulomatous"[All Fields])) OR ("thyroiditis, subacute"[MeSH Terms] OR ("thyroiditis"[All Fields] AND "subacute"[All Fields]) OR "subacute thyroiditis"[All Fields] OR ("thyroiditis"[All Fields] AND "granulomatous"[All Fields]) OR "thyroiditis granulomatous"[All Fields]) OR ("thyroiditis, subacute"[MeSH Terms] OR ("thyroiditis"[All Fields] AND "subacute"[All Fields]) OR "subacute thyroiditis"[All Fields] OR ("subacute"[All Fields] AND "nonsuppurative"[All Fields] AND "thyroiditis"[All Fields]) OR "subacute nonsuppurative thyroiditis"[All Fields]) OR ("thyroiditis, subacute"[MeSH Terms] OR ("thyroiditis"[All Fields] AND "subacute"[All Fields]) OR "subacute thyroiditis"[All Fields] OR ("nonsuppurative"[All Fields] AND "thyroiditides"[All Fields] AND "subacute"[All Fields])) OR ("thyroiditis, subacute"[MeSH Terms] OR ("thyroiditis"[All Fields] AND "subacute"[All Fields]) OR "subacute thyroiditis"[All Fields] OR ("nonsuppurative"[All Fields] AND "thyroiditis"[All Fields] AND "subacute"[All Fields])) OR ("thyroiditis, subacute"[MeSH Terms] OR ("thyroiditis"[All Fields] AND "subacute"[All Fields]) OR "subacute thyroiditis"[All Fields] OR ("subacute"[All Fields] AND "nonsuppurative"[All Fields] AND "thyroiditides"[All Fields])) OR ("thyroiditis, subacute"[MeSH Terms] OR ("thyroiditis"[All Fields] AND "subacute"[All Fields]) OR "subacute thyroiditis"[All Fields] OR ("thyroiditides"[All Fields] AND "subacute"[All Fields] AND "nonsuppurative"[All Fields])) OR ("thyroiditis, subacute"[MeSH Terms] OR ("thyroiditis"[All Fields] AND "subacute"[All Fields]) OR "subacute thyroiditis"[All Fields] OR ("thyroiditis"[All Fields] AND "subacute"[All Fields] AND "nonsuppurative"[All Fields]) OR "thyroiditis subacute nonsuppurative"[All Fields]) OR ("thyroiditis, subacute"[MeSH Terms] OR ("thyroiditis"[All Fields] AND "subacute"[All Fields]) OR "subacute thyroiditis"[All Fields] OR ("de"[All Fields] AND "quervain"[All Fields] AND "thyroiditis"[All Fields]) OR "de quervain thyroiditis"[All Fields]) OR ("thyroiditis, subacute"[MeSH Terms] OR ("thyroiditis"[All Fields] AND "subacute"[All Fields]) OR "subacute thyroiditis"[All Fields] OR ("thyroiditis"[All Fields] AND "de"[All Fields] AND "quervain"[All Fields]) OR "thyroiditis de quervain"[All Fields]) OR ("thyroiditis, subacute"[MeSH Terms] OR ("thyroiditis"[All Fields] AND "subacute"[All Fields]) OR "subacute thyroiditis"[All Fields] OR ("giant"[All Fields] AND "cell"[All Fields] AND "thyroiditis"[All Fields]) OR "giant cell thyroiditis"[All Fields]) OR ("thyroiditis, subacute"[MeSH Terms] OR ("thyroiditis"[All Fields] AND "subacute"[All Fields]) OR "subacute thyroiditis"[All Fields] OR ("cell"[All Fields] AND "thyroiditides"[All Fields] AND "giant"[All Fields])) OR ("thyroiditis, subacute"[MeSH Terms] OR ("thyroiditis"[All Fields] AND "subacute"[All Fields]) OR "subacute thyroiditis"[All Fields] OR ("cell"[All Fields] AND "thyroiditis"[All Fields] AND "giant"[All Fields])) OR ("thyroiditis, subacute"[MeSH Terms] OR ("thyroiditis"[All Fields] AND "subacute"[All Fields]) OR "subacute thyroiditis"[All Fields] OR ("giant"[All Fields] AND "cell"[All Fields] AND "thyroiditides"[All Fields])) OR ("thyroiditis, subacute"[MeSH Terms] OR ("thyroiditis"[All Fields] AND "subacute"[All Fields]) OR "subacute thyroiditis"[All Fields] OR ("thyroiditides"[All Fields] AND "giant"[All Fields] AND "cell"[All Fields])) OR ("thyroiditis, subacute"[MeSH Terms] OR ("thyroiditis"[All Fields] AND "subacute"[All Fields]) OR "subacute thyroiditis"[All Fields] OR ("thyroiditis"[All Fields] AND "giant"[All Fields] AND "cell"[All Fields]) OR "thyroiditis giant cell"[All Fields]))) AND ("Glucocorticoids"[MeSH Terms] OR ("Glucocorticoids"[Pharmacological Action] OR "Glucocorticoids"[MeSH Terms] OR "Glucocorticoids"[All Fields] OR "glucocorticoid"[All Fields] OR ("Glucocorticoids"[Pharmacological Action] OR "Glucocorticoids"[MeSH Terms] OR "Glucocorticoids"[All Fields] OR ("glucocorticoid"[All Fields] AND "effect"[All Fields]) OR "glucocorticoid effect"[All Fields]) OR ("Glucocorticoids"[Pharmacological Action] OR "Glucocorticoids"[MeSH Terms] OR "Glucocorticoids"[All Fields] OR ("effect"[All Fields] AND "glucocorticoid"[All Fields]) OR "effect glucocorticoid"[All Fields]) OR ("Glucocorticoids"[Pharmacological Action] OR "Glucocorticoids"[MeSH Terms] OR "Glucocorticoids"[All Fields] OR ("glucorticoid"[All Fields] AND "effects"[All Fields]) OR "glucorticoid effects"[All Fields]) OR ("Glucocorticoids"[Pharmacological Action] OR "Glucocorticoids"[MeSH Terms] OR "Glucocorticoids"[All Fields] OR ("effects"[All Fields] AND "glucorticoid"[All Fields])))) AND ("anti inflammatory agents, non steroidal"[MeSH Terms] OR ("anti inflammatory agents non steroidal"[Pharmacological Action] OR "anti inflammatory agents, non steroidal"[MeSH Terms] OR ("anti inflammatory"[All Fields] AND "agents"[All Fields] AND "non steroidal"[All Fields]) OR "non-steroidal anti-inflammatory agents"[All Fields] OR "nsaid"[All Fields] OR "nsaids"[All Fields] OR "nsaid s"[All Fields] OR ("anti inflammatory agents non steroidal"[Pharmacological Action] OR "anti inflammatory agents, non steroidal"[MeSH Terms] OR ("anti inflammatory"[All Fields] AND "agents"[All Fields] AND "non steroidal"[All Fields]) OR "non-steroidal anti-inflammatory agents"[All Fields] OR ("nonsteroidal"[All Fields] AND "anti"[All Fields] AND "inflammatory"[All Fields] AND "agent"[All Fields]) OR "nonsteroidal anti inflammatory agent"[All Fields]) OR ("anti inflammatory agents non steroidal"[Pharmacological Action] OR "anti inflammatory agents, non steroidal"[MeSH Terms] OR ("anti inflammatory"[All Fields] AND "agents"[All Fields] AND "non steroidal"[All Fields]) OR "non-steroidal anti-inflammatory agents"[All Fields] OR ("agent"[All Fields] AND "nonsteroidal"[All Fields] AND "anti"[All Fields] AND "inflammatory"[All Fields]) OR "agent nonsteroidal anti inflammatory"[All Fields]) OR ("anti inflammatory agents non steroidal"[Pharmacological Action] OR "anti inflammatory agents, non steroidal"[MeSH Terms] OR ("anti inflammatory"[All Fields] AND "agents"[All Fields] AND "non steroidal"[All Fields]) OR "non-steroidal anti-inflammatory agents"[All Fields] OR ("anti"[All Fields] AND "inflammatory"[All Fields] AND "agent"[All Fields] AND "nonsteroidal"[All Fields])) OR ("anti inflammatory agents non steroidal"[Pharmacological Action] OR "anti inflammatory agents, non steroidal"[MeSH Terms] OR ("anti inflammatory"[All Fields] AND "agents"[All Fields] AND "non steroidal"[All Fields]) OR "non-steroidal anti-inflammatory agents"[All Fields] OR ("nonsteroidal"[All Fields] AND "anti"[All Fields] AND "inflammatory"[All Fields] AND "agent"[All Fields]) OR "nonsteroidal anti inflammatory agent"[All Fields]) OR ("anti inflammatory agents non steroidal"[Pharmacological Action] OR "anti inflammatory agents, non steroidal"[MeSH Terms] OR ("anti inflammatory"[All Fields] AND "agents"[All Fields] AND "non steroidal"[All Fields]) OR "non-steroidal anti-inflammatory agents"[All Fields] OR "nsaid"[All Fields] OR "nsaids"[All Fields] OR "nsaid s"[All Fields]) OR ("anti inflammatory agents non steroidal"[Pharmacological Action] OR "anti inflammatory agents, non steroidal"[MeSH Terms] OR ("anti inflammatory"[All Fields] AND "agents"[All Fields] AND "non steroidal"[All Fields]) OR "non-steroidal anti-inflammatory agents"[All Fields] OR ("antiinflammatory"[All Fields] AND "agents"[All Fields] AND "non"[All Fields] AND "steroidal"[All Fields])) OR ("anti inflammatory agents non steroidal"[Pharmacological Action] OR "anti inflammatory agents, non steroidal"[MeSH Terms] OR ("anti inflammatory"[All Fields] AND "agents"[All Fields] AND "non steroidal"[All Fields]) OR "non-steroidal anti-inflammatory agents"[All Fields] OR ("antiinflammatory"[All Fields] AND "agents"[All Fields] AND "nonsteroidal"[All Fields]) OR "antiinflammatory agents nonsteroidal"[All Fields]) OR ("anti inflammatory agents non steroidal"[Pharmacological Action] OR "anti inflammatory agents, non steroidal"[MeSH Terms] OR ("anti inflammatory"[All Fields] AND "agents"[All Fields] AND "non steroidal"[All Fields]) OR "non-steroidal anti-inflammatory agents"[All Fields] OR ("nonsteroidal"[All Fields] AND "antiinflammatory"[All Fields] AND "agents"[All Fields]) OR "nonsteroidal antiinflammatory agents"[All Fields]) OR ("anti inflammatory agents non steroidal"[Pharmacological Action] OR "anti inflammatory agents, non steroidal"[MeSH Terms] OR ("anti inflammatory"[All Fields] AND "agents"[All Fields] AND "non steroidal"[All Fields]) OR "non-steroidal anti-inflammatory agents"[All Fields] OR ("non"[All Fields] AND "steroidal"[All Fields] AND "anti"[All Fields] AND "inflammatory"[All Fields] AND "agents"[All Fields]) OR "non steroidal anti inflammatory agents"[All Fields]) OR ("anti inflammatory agents non steroidal"[Pharmacological Action] OR "anti inflammatory agents, non steroidal"[MeSH Terms] OR ("anti inflammatory"[All Fields] AND "agents"[All Fields] AND "non steroidal"[All Fields]) OR "non-steroidal anti-inflammatory agents"[All Fields] OR ("non"[All Fields] AND "steroidal"[All Fields] AND "anti"[All Fields] AND "inflammatory"[All Fields] AND "agents"[All Fields]) OR "non steroidal anti inflammatory agents"[All Fields]) OR ("anti inflammatory agents non steroidal"[Pharmacological Action] OR "anti inflammatory agents, non steroidal"[MeSH Terms] OR ("anti inflammatory"[All Fields] AND "agents"[All Fields] AND "non steroidal"[All Fields]) OR "non-steroidal anti-inflammatory agents"[All Fields] OR ("nonsteroidal"[All Fields] AND "anti"[All Fields] AND "inflammatory"[All Fields] AND "agents"[All Fields]) OR "nonsteroidal anti inflammatory agents"[All Fields]) OR ("anti inflammatory agents non steroidal"[Pharmacological Action] OR "anti inflammatory agents, non steroidal"[MeSH Terms] OR ("anti inflammatory"[All Fields] AND "agents"[All Fields] AND "non steroidal"[All Fields]) OR "non-steroidal anti-inflammatory agents"[All Fields] OR ("nonsteroidal"[All Fields] AND "anti"[All Fields] AND "inflammatory"[All Fields] AND "agents"[All Fields]) OR "nonsteroidal anti inflammatory agents"[All Fields]) OR ("anti inflammatory agents non steroidal"[Pharmacological Action] OR "anti inflammatory agents, non steroidal"[MeSH Terms] OR ("anti inflammatory"[All Fields] AND "agents"[All Fields] AND "non steroidal"[All Fields]) OR "non-steroidal anti-inflammatory agents"[All Fields] OR ("non"[All Fields] AND "steroidal"[All Fields] AND "anti"[All Fields] AND "inflammatory"[All Fields] AND "agent"[All Fields]) OR "non steroidal anti inflammatory agent"[All Fields]) OR ("anti inflammatory agents non steroidal"[Pharmacological Action] OR "anti inflammatory agents, non steroidal"[MeSH Terms] OR ("anti inflammatory"[All Fields] AND "agents"[All Fields] AND "non steroidal"[All Fields]) OR "non-steroidal anti-inflammatory agents"[All Fields] OR ("agent"[All Fields] AND "non"[All Fields] AND "steroidal"[All Fields] AND "anti"[All Fields] AND "inflammatory"[All Fields]) OR "agent non steroidal anti inflammatory"[All Fields]) OR ("anti inflammatory agents non steroidal"[Pharmacological Action] OR "anti inflammatory agents, non steroidal"[MeSH Terms] OR ("anti inflammatory"[All Fields] AND "agents"[All Fields] AND "non steroidal"[All Fields]) OR "non-steroidal anti-inflammatory agents"[All Fields] OR ("anti"[All Fields] AND "inflammatory"[All Fields] AND "agent"[All Fields] AND "non"[All Fields] AND "steroidal"[All Fields]) OR "anti inflammatory agent non steroidal"[All Fields]) OR ("anti inflammatory agents non steroidal"[Pharmacological Action] OR "anti inflammatory agents, non steroidal"[MeSH Terms] OR ("anti inflammatory"[All Fields] AND "agents"[All Fields] AND "non steroidal"[All Fields]) OR "non-steroidal anti-inflammatory agents"[All Fields] OR ("non"[All Fields] AND "steroidal"[All Fields] AND "anti"[All Fields] AND "inflammatory"[All Fields] AND "agent"[All Fields]) OR "non steroidal anti inflammatory agent"[All Fields]) OR ("anti inflammatory agents non steroidal"[Pharmacological Action] OR "anti inflammatory agents, non steroidal"[MeSH Terms] OR ("anti inflammatory"[All Fields] AND "agents"[All Fields] AND "non steroidal"[All Fields]) OR "non-steroidal anti-inflammatory agents"[All Fields] OR ("anti"[All Fields] AND "inflammatory"[All Fields] AND "agents"[All Fields] AND "nonsteroidal"[All Fields]) OR "anti inflammatory agents nonsteroidal"[All Fields]) OR ("anti inflammatory agents non steroidal"[Pharmacological Action] OR "anti inflammatory agents, non steroidal"[MeSH Terms] OR ("anti inflammatory"[All Fields] AND "agents"[All Fields] AND "non steroidal"[All Fields]) OR "non-steroidal anti-inflammatory agents"[All Fields] OR ("analgesics"[All Fields] AND "anti"[All Fields] AND "inflammatory"[All Fields]) OR "analgesics anti inflammatory"[All Fields]) OR ("anti inflammatory agents non steroidal"[Pharmacological Action] OR "anti inflammatory agents, non steroidal"[MeSH Terms] OR ("anti inflammatory"[All Fields] AND "agents"[All Fields] AND "non steroidal"[All Fields]) OR "non-steroidal anti-inflammatory agents"[All Fields] OR ("anti"[All Fields] AND "inflammatory"[All Fields] AND "analgesics"[All Fields]) OR "anti inflammatory analgesics"[All Fields]) OR ("anti inflammatory agents non steroidal"[Pharmacological Action] OR "anti inflammatory agents, non steroidal"[MeSH Terms] OR ("anti inflammatory"[All Fields] AND "agents"[All Fields] AND "non steroidal"[All Fields]) OR "non-steroidal anti-inflammatory agents"[All Fields] OR ("aspirin"[All Fields] AND "like"[All Fields] AND "agents"[All Fields]) OR "aspirin like agents"[All Fields]) OR ("anti inflammatory agents non steroidal"[Pharmacological Action] OR "anti inflammatory agents, non steroidal"[MeSH Terms] OR ("anti inflammatory"[All Fields] AND "agents"[All Fields] AND "non steroidal"[All Fields]) OR "non-steroidal anti-inflammatory agents"[All Fields] OR ("aspirin"[All Fields] AND "like"[All Fields] AND "agents"[All Fields]) OR "aspirin like agents"[All Fields]) OR ("anti inflammatory agents non steroidal"[Pharmacological Action] OR "anti inflammatory agents, non steroidal"[MeSH Terms] OR ("anti inflammatory"[All Fields] AND "agents"[All Fields] AND "non steroidal"[All Fields]) OR "non-steroidal anti-inflammatory agents"[All Fields] OR ("aspirin"[All Fields] AND "like"[All Fields] AND "agent"[All Fields]) OR "aspirin like agent"[All Fields]) OR ("anti inflammatory agents non steroidal"[Pharmacological Action] OR "anti inflammatory agents, non steroidal"[MeSH Terms] OR ("anti inflammatory"[All Fields] AND "agents"[All Fields] AND "non steroidal"[All Fields]) OR "non-steroidal anti-inflammatory agents"[All Fields] OR ("agent"[All Fields] AND "aspirin"[All Fields] AND "like"[All Fields])) OR ("anti inflammatory agents non steroidal"[Pharmacological Action] OR "anti inflammatory agents, non steroidal"[MeSH Terms] OR ("anti inflammatory"[All Fields] AND "agents"[All Fields] AND "non steroidal"[All Fields]) OR "non-steroidal anti-inflammatory agents"[All Fields] OR ("aspirin"[All Fields] AND "like"[All Fields] AND "agent"[All Fields]) OR "aspirin like agent"[All Fields])))
